# Evaluation of the Final Adult Height and Its Determinants in Patients with Growth Hormone Deficiency: A Single-centre Experience from the South-Eastern Region of Turkey

**DOI:** 10.4274/jcrpe.galenos.2020.2019.0218

**Published:** 2020-09-02

**Authors:** Meliha Demiral, Edip Unal, Birsen Baysal, Rıza Taner Baran, Hüseyin Demirbilek, Mehmet Nuri Özbek

**Affiliations:** 1 Gazi Yaşargil Training and Research Hospital, Clinic of Paediatric Endocrinology, Diyarbakır, Turkey; 2 Gazi Yaşargil Training and Research Hospital, Clinic of Paediatrics, Diyarbakır, Turkey; 3 Antalya Training and Research Hospital, Clinic of Paediatric Endocrinology, Antalya, Turkey; 4 Hacettepe University Faculty of Medicine, Department of Paediatric Endocrinology, Ankara, Turkey

**Keywords:** Isolated growth hormone deficiency, multiple pituitary hormone deficiency, growth hormone treatment, final height, puberty

## Abstract

**Objective:**

The aim was to determine the final adult height (FAH) achieved by recombinant human growth hormone (rhGH) treatment, the factors affecting FAH and the success of attaining the genetic potential.

**Methods:**

Data of 133 patients treated with rhGH therapy were reviewed retrospectively. Patients were grouped according to diagnosis, either isolated GH deficiency (IGHD) or multiple pituitary hormone deficiency (MPHD), and by sex, and pubertal status at the beginning of treatment.

**Results:**

The mean age of initiation of treatment was 12.3±2.18 years, and the mean duration of rhGH treatment was 3.65±1.5 years. The mean height standard deviation score (SDS) at diagnosis was -3.11±0.75 SD. All patients received a standardized GH dose of 0.033 mg/kg/day. Mean FAH-SDS was -1.8±0.77 and delta height-SDS (the change in height SDS between the beginning and end of treatment) was 1.28±0.94 SD. FAH-SDS was -1.79±0.86 SD in males; -1.82±0.64 in females (p=0.857); -1.94±0.71 at the beginning of treatment in pubertal patients and -1.68±0.81 in prepubertal patients (p=0.056); -1.84±0.89 in patients with IGHD and -0.47±0.2 in patients with MPHD (p˃0.05). In multiple regression analysis, First year delta height-SDS was the most predictive factor for both FAH-SDS and delta height-SDS.

**Conclusion:**

The majority of our patients achieved a final height compatible with their genetic potential as well as population standards when treated with rhGH even having started at a relatively late age. First year delta height-SDS was a predictive factor for FAH.

**What is already known on this topic?**

Patients who the growth hormone (GH) treatment is started in the prepubertal period are known to achieve better final adult height (FAH) standard deviation score (SDS) and delta height SDS than those started in the pubertal period.

**What this study adds?**

The present study provides the data for height outcome of a large series of patients who reached the final height with GH therapy in a single center using a fixed dose GH therapy regimen. There was no difference between pubertal and prepubertal patients for FAH-SDS and delta height SDS with this fixed dose GH therapy. Commencement of GH therapy in the prepubertal period was not found to be associated with a better height outcome.

## Introduction

The goal of growth hormone (GH) therapy is to increase the growth rate in the short term and to improve the final height which, in turn, affects the psychosocial status of the person in the long-term (1). GH also has a positive effect on cardiac function, skeletal structure and body composition due to its impact on bone, lipid, protein and glucose metabolism (2). With the introduction of recombinant GH (rhGH), GH treatment has begun to be applied in a growing number of cases of GH deficiency (GHD), as well as in various diseases with short stature other than GHD (3). Chronic renal failure, Turner syndrome, small for gestational age birth history, idiopathic short stature, SHOX gene mutation, and Prader Willi syndrome are indicated for using different doses of GH (4).

GH is administered subcutaneously with a dose of 0.025-0.1 mg/kg/day (3,5) for six or seven days per week until the final adult height (FAH) is achieved. Factors affecting response to GH therapy have been reported to be frequency, dose, duration of treatment, adherence to treatment, age at onset of treatment, birth length, height standard deviation score (SDS) at the beginning of treatment, parental height, and the first-year response to GH treatment (6,7,8). The growth rate is particularly high in the pubertal period, but there is limited time available for rhGH treatment in pubertal patients. Therefore, a higher dose of rhGH is recommended for patients during puberty (9,10). The aim of the present study was to determine the FAH, factors affecting FAH and the genetic potential of FAH in a group of patients with both isolated GH deficiency (IGHD) and multiple pituitary hormone deficiency (MPHD) with rhGH treatment at a standardized dose of 0.033 mg/kg/day.

## Methods

### Study Population

**Figure 1:**
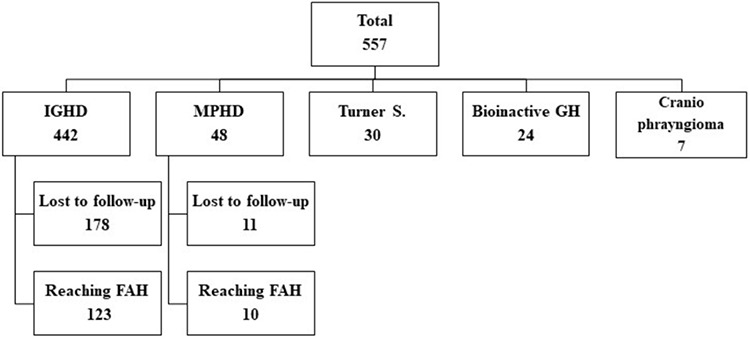
A flowchart of patients with a diagnosis of growth hormone (GH) deficiency who received GH treatment and their follow up GH: growth hormone, IGHD: idiopathic GH deficiency, MPHD: multiple pituitary hormone deficiency, FAH: final adult height

In the present study, we recruited 133 patients with IGHD and MPHD who reached FAH from among the 557 patients who had received rhGH treatment in the Pediatric Endocrinology Department of Gazi Yaşargil Training and Research Hospital, a tertiary pediatric endocrine centre, between 2010 and 2018 (Figure 1). The study was performed in accordance with the rules of Declaration of Helsinki and approved by the Institutional Ethics Committee of Gazi Yaşargil Training and Research Hospital (document number: 4.7.2019/7305). The clinical and laboratory findings of the patients were retrospectively reviewed from hospital files.

GH initiation criteria were; to have a height below -2 SD, an annual growth velocity below 25^th^ percentile for age and sex, a GH peak below 10 ng/mL on two GH provocation tests (clonidine and L-dopa), bone age retarded more than 2 SD in prepubertal patients and having open epiphysis in pubertal patients (11). Patients who had skeletal dysplasia, chromosomal disorder including Turner syndrome and Noonan syndrome, systemic disease, intracranial tumour, surgical intervention, radiotherapy and hormone deficiency secondary to chemotherapy were excluded. The patients were grouped into IGHD and MPHD according to the diagnosis and were also grouped into those who started treatment in the prepubertal and pubertal periods. All patients received subcutaneous 0.033 mg/kg/day GH treatment every day. In patients with MPHD, other deficient hormones were also replaced. Puberty was defined as breast development ≥ Tanner stage 2 in girls and testicular volume ≥4 mL in boys. Testicular volume was evaluated using Prader orchidometer. According to the rules determined by the social security institution in our country, GH treatment is discontinued when the height reaches 155 cm in girls and 165 cm in boys. Besides, during the follow-up of GH treatment, patients who had a height velocity less than 2 cm in nine months and/or whose chronological age was greater than 17 and/or a bone age greater than 14 in females and 15 in males had their GH treatment stopped (6). FAH-SDS, mid parental height (MPH) (mean parental height±6.5 cm) of all patients were calculated according to growth charts developed for Turkish children (12). Predicted adult height (PAH) SDS was calculated according to the Bayley-Pinneau method for children older than seven years and the Roche-Wainer-Thissen methods for children younger than seven years. Parent-specific lower limit of height SDS was calculated as (0.5˟ MPHSDS) -1.73 (13). Delta height SDS was the difference between height SDS at the beginning of the treatment and FAH-SDS. First-year delta height SDS was the difference between height SDS at the beginning of treatment and height SDS at the end of the first year of treatment.

### Statistical Analysis

The Statistical Package for the Social Sciences, version 24.0, (IBM Inc., Armonk, NY, USA) was used for statistical analyses. Data were displayed as mean±SD or median [25-75 interquartile range (IQR)]. Kolmogorov-Smirnov and Shapiro-Wilk tests were used for normality distribution of the data. In order to compare the data, an independent sample t-test was used in the normally distributed groups, and non-parametric tests were used in the non-normally distributed groups. To evaluate the relationship between FAH-SDS and delta height SDS with PAH-SDS, MPH-SDS, first year delta height SDS, treatment duration, age and GHD diagnostic parameters [height SDS, weight SDS, body mass index (BMI) SDS, GH peak, insulin-like growth factor-1 (IGF1) SDS, IGF binding protein-3 (IGFBP3) SDS] Pearson correlation analysis and multiple regression analysis was performed. A p value <0.05 was considered statistically significant.

## Results

A total of 133 patients (54 females; 40.6%) were included in the study. At the beginning of the treatment, 63 (47.4%) of the patients were pubertal and 70 were prepubertal. In addition 123 (92.5%) had IGHD, and 10 (7.5%) had MPHD. Birth weight was lower than -2 SDS in 15 patients. Pretreatment mean height SDS was -3.11±0.75 and ranged from -6.1 to -1.71 SD. Height SDS was below -2 SD in all patients except for a patient with MPHD who had a height SDS of -1.71, who also suffered from recurrent hypoglycemia attributed to the GH deficiency which resolved after rhGH commencement. All patients with a height-adjusted weight below -2 SDS and/or BMI-SDS <-2 were also evaluated for protein-energy malnutrition. In our study, 85 of the patients were underweight according to height-adjusted weight SDS and 16 had BMI-SDS <-2. These patients were followed for at least a one year period with appropriate calorie intake. Patients who had low IGF SDS and low growth velocity in the first year of follow-up, despite appropriate calorie intake, were evaluated for GHD. Mean peak GH in the GH stimulation test was 5.32±2.41 ng/mL which ranged from 0.2 to 9.8 SD, mean IGF SDS was -1.2±1.04 and ranged from -3.4 to 2.1 SD. In total 80.4% of the patients were above the parent-specific lower limit for final height and 70% were within the normal range according to the population. Anthropometric measurements, laboratory values, and comparisons between the groups are shown in Table 1. The mean age of onset of GH treatment and the mean bone age was lower in girls than in boys (p<0.001) (Table 1). There was no statistically significant difference between boys and girls in height SDS, weight SDS, BMI-SDS, GH peak, FAH-SDS, MPH-SDS, PAH-SDS, and delta height SDS (the change in height SDS between the beginning and end of treatment) (Table 1).

**Figure 2:**
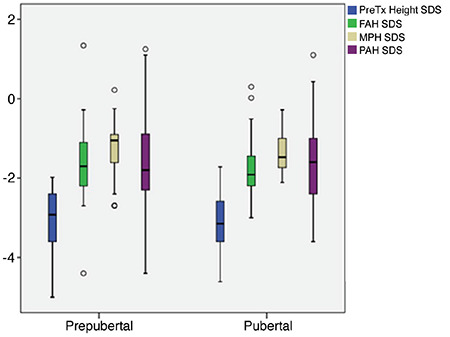
Comparison of final adult height and predictive variables outcomes in prepubertal and pubertal groups (lines within the boxes indicate the median, the limits of the boxes indicate the 25^th^ and 75^th^ percentiles, and the extensions of the boxes indicate the minimum and maximum) FAH: final adult height, MPH: multiple pituitary hormone, PAH: predicted adult height, SDS: standart deviation score, Tx: treatment

At the time of the treatment, age and bone age of pubertal patients were significantly higher than those of prepubertal patients (p<0.001) (Table 1). There was no statistically significant difference between the pre-pubertal and pubertal groups in terms of height SDS, weight SDS, BMI-SDS, peak GH, FAH-SDS, MPH-SDS, PAH-SDS and delta height SDS (Table 1) (Figure 2).

**Figure 3:**
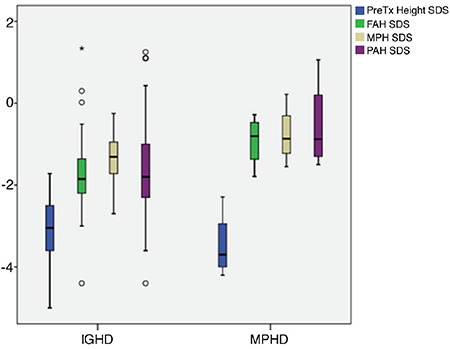
Comparison of final adult height and predictive variables outcomes in idiopathic growth hormone deficiency and multiple pituitary hormone deficiency groups (lines within the boxes indicate the median, the limits of the boxes indicate the 25^th^ and 75^th^ percentiles, and the extensions of the boxes indicate the minimum and maximum) FAH: final adult height, MPH: multiple pituitary hormone, PAH: predicted adult height, SDS: standart deviation score, Tx: treatment, IGHD: idiopathic growth hormone deficiency, MPHD: multiple pituitary hormone deficiency

GH peak, IGF1 SDS, IGFBP3 SDS, PAH-SDS were significantly lower in patients with MPHD compared to those with IGHD (Table 1). There was no statistically significant difference between the two groups for height SDS, weight SDS, duration of treatment, FAH-SDS, MPH-SDS, delta height SDS (Table 1) (Figure 3).

IGF1 levels were below -2 SDS in 25 patients, initially. There was no statistically significant difference between the FAH-SDS of patients who had an IGF1 below or above -2 SDS. However, delta height SDS was higher in the group with an IGF1 below -2 SDS.

FAH-SDS had a negative correlation with age and bone age at the beginning of treatment, and a positive correlation with height, weight, and first-year delta height SDS. Delta height SDS was negatively correlated with bone age, height, and weight, IGF SDS, IGFBP3 SDS, and positively correlated with first-year delta height SDS and treatment duration (Table 2). In multiple regression analysis, PAH-SDS, weight SDS, first year delta height SDS were predictive factors for both FAH-SDS and delta height SDS. A first year 1 SD increase in delta height SDS was found to be associated with a 0.68 SD increase in FAH-SDS (35% of variability, 0.18 of error SD) and 0.63 SD increase in delta height SDS (52% of variability, 0.2 of error SD). Besides, the duration of treatment and bone age at the time of the diagnosis was associated with FAH-SDS, whereas they were not associated with delta height SDS (Table 3).

## Discussion

In the present study, the FAH achieved with GH therapy was evaluated and the factors affecting FAH in a group of patients who received 0.033 mg/kg/day GH treatment for IGHD and MPHD every day were investigated. Patients with MPHD had achieved a better final height (mean FAH-SDS was -0.47 SD) compared to cases with isolated IGHD (mean FAH-SDS was -1.84 SD).

In studies conducted in our country, by Yordam et al (14) and Kurnaz et al (15), FAH-SDS was reported to be -2.06 and -1.8 respectively, in patients with IGHD who received GH therapy with similar duration and treatment doses (Table 3). In an international study in 1619 patients with IGHD the FAH-SDS was reported to be -1.4 SD (6). Cappa et al (16), Rachmiel et al (17), Straetemans et al (18), Carel et al (19), and Thomas et al (20) reported -0.86, -1.04, -1.74, -1.6, -0.8 FAH-SDS in patients with IGHD, respectively. In all these studies, although the GH treatment dose was similar or lower than our study in IGHD groups, FAH-SDS were better than in our study, and delta height SDS was similar (Table 4). In these studies, the age of onset of treatment was earlier and the duration of treatment was longer. In our country, the duration of treatment is shorter due to the discontinuation of treatment when the height reaches 155 cm in females and 165 cm in males as a rule determined by social security institution. Better response with lower treatment doses in other studies suggested that treatment dose may not an essential factor affecting FAH-SDS (8). However, the average age of treatment was earlier in these studies which may achieved a better response with lower doses. In patients with MPHD, treatment response and final height have been reported to be better than in patients with IGHD (15,21). However, although the treatment response to MPHD is better, FAH-SDS can be similar to that seen in patients with IGHD (GHD) due to more severe GHD, shorter initial height, and the presence of other hormone deficiencies accompanying GHD (22). In agreement with previous reports, both delta height SDS and FAH-SDS were higher in patients with MPHD than patients with IGHD (6,14,15,16,18). This was attributed to younger age for the onset of the treatment, longer duration of treatment, lower bone age, and lower IGF1 SDS as well as peak GH values in the GH stimulation test. IGF1 SDS and peak GH value in GH stimulation test were lower in patients with MPHD (p<0.05). However, the difference between delta height SDS and FAH-SDS had not reached a statistical significance, presumably due to the small number of patients with MPHD.

Although early diagnosis and treatment with GH can provide a final height consistent with MPH, patients’ final height usually remained below the average of the population (6,17). Similarly, in our study, although 80.4% of the patients had reached a final height above parent-specific lower limits, 70% of the patients had a FAH-SDS above -2 SD according to the growth charts determined for the Turkish population. The rate of achieving a FAH consistent with genetic potential has been reported from to be between 81% and 92% (6,15,17,20). The factors affecting the achievement of final height compatible with genetic potential have been reported to include treatment compliance, treatment dose and age of initiation. MPH is one of the important factors affecting the final height in children receiving GH treatment. Treatment response in GHD patients with short parents was also low (23). However, it is important to distinguish between normal children with genetic short stature and patients with GHD whose parents are short. In our study, mean MPH-SDS was -1.3, which was achieved in 80% of the patients diagnosed with IGHD. In studies evaluating the GH treatment response, the mean FAH-MPH has been reported to be between 0.0 and -0.6 SD (6,14,15,16,17,18,19,20). In our study, FAH-MPH-SDS was -0.43 SD in IGHD and -0.15 SD in MPHD, and the findings were consistent with the literature.

About half of our patients were at an age at which puberty would normally be expected to start when GH treatment was started. Although the chronological age and bone age were higher in the pubertal group and the duration of GH treatment was longer in the prepubertal group, there was no statistically significant difference between the two groups in terms of FAH-SDS and delta height SDS. Similar to our study, Kurnaz et al (15) did not detect a difference between FAH-SDS in pubertal and prepubertal patients but found that delta height SDS was higher in pubertal patients. Cacciari et al (24) reported that pubertal patients had greater SDS gain than prepubertal patients (24). This suggests that GH therapy initiated in puberty has a synergistic effect with the pubertal growth spurt, resulting in a better treatment response than expected. These results indicated that apart from growth factors, growth during puberty may be affected by other individual factors such as the age of onset and rate of pubertal progression (25). However, appropriate GH treatment, even when initiated at the pubertal ages, could help to achieve a final height compatible with genetic height potential.

In our study, PAH-SDS, weight SDS, and first year delta height SDS were predictive factors for both FAH-SDS and delta height SDS. Duration of treatment and bone age were the only predictive factors for FAH-SDS. Among these parameters, the most significant factor in predicting height gain at the end of the treatment was first-year delta height SDS. First year response of GH treatment, is one of the most important predictor of FAH (15,26). During the first year of GH treatment, 1 SD increase in delta height SDS was associated with 0.68 SD increase in FAH-SDS and 0.63 SD increase in delta height SDS in our study. In a study; first year responsiveness was the second relevant predictors of near adult height in IGHD and MPHD (coefficient B: 0.4 and 0.3 respectively) (6).

MPH-SDS has been reported as an important predictor of final height and patients with short parents have a poorer response to treatment (6). In our case, MPH-SDS was not associated with either FAH-SDS or delta height SDS. The high rate of consanguineous marriages in our region may suggest that familial GHD may also be common. Therefore, although we could not evaluate the parents in terms of GHD, achieving a better final height compared to their genetic potential in our patients can be attributed to a high rate of missed GHD in the parents.

### Study Limitations

The present study has some limitations which may affect the FAH and delta FAH-SDS. About half of the 557 patients with GH deficiency and GH treatment who had been referred to our clinic from other centres but had become lost to regular follow-up. Therefore, the number of patients who reached FAH was low (Figure 1). Moreover, in the majority of cases, the treatment had to be discontinued before achieving the maximum achievable final height, due to the rules of the social security which funds GH therapy, that the final height of 155 cm for girls and 165 cm for boys were assigned as criteria for stopping the treatment. In addition, we could not evaluate height gain before and after puberty separately in patients in whom GH treatment had been commenced in the prepubertal period.

## Conclusion

In conclusion, the majority of our patients have achieved a final height compatible with their genetic potential and population standards even starting at a later age. First year delta height SDS was found to be the most predictive factor for FAH. Interestingly, the commencement of GH therapy in the prepubertal period was not found to be associated with a better height outcome. Recognition of these factors and individualization of the treatment accordingly will help to optimize the long-term response to GH treatment.

## Figures and Tables

**Table 1:**
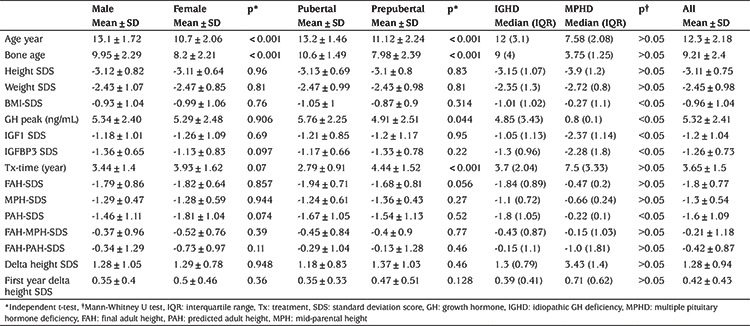
Comparison between groups

**Table 2:**
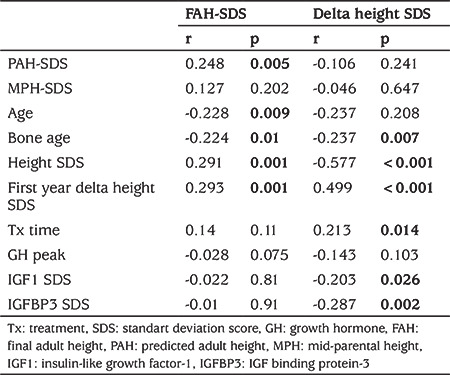
Correlation between final adult height standart deviation score (SDS), delta height SDS and other parameters

**Table 3:**
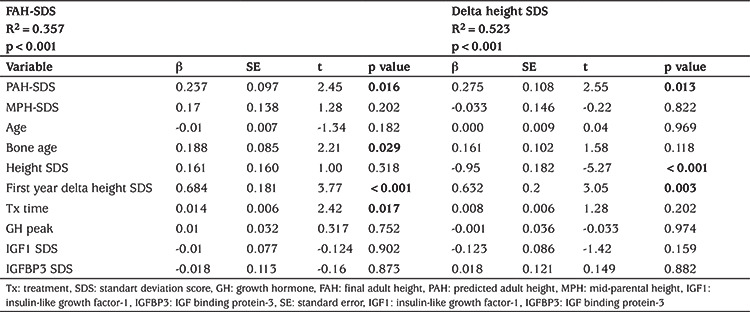
Multiple linear regression analysis on final adult height standart deviation score (SDS) and delta height SDS

**Table 4:**
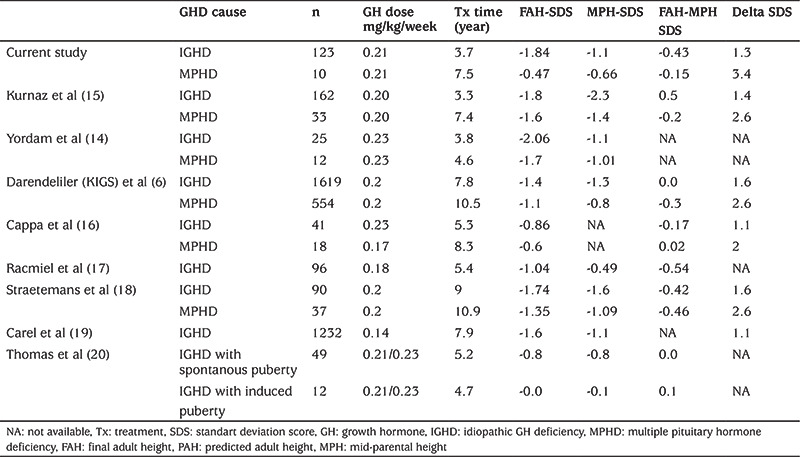
Comparison of final adult height outcome in previously reported studies
